# Serum 25(OH) Vitamin D Levels in Polish Women during Pregnancies Complicated by Hypertensive Disorders and Gestational Diabetes

**DOI:** 10.3390/ijms17101574

**Published:** 2016-09-27

**Authors:** Piotr Domaracki, Pawel Sadlecki, Grazyna Odrowaz-Sypniewska, Ewa Dzikowska, Pawel Walentowicz, Joanna Siodmiak, Marek Grabiec, Malgorzata Walentowicz-Sadlecka

**Affiliations:** 1 Department of Obstetrics and Gynecology, L. Rydygier Collegium in Bydgoszcz, Nicolaus Copernicus University, ul. Ujejskiego 75, Bydgoszcz 85-168, Poland; domarackipiotr@gmail.com (P.D.); pawelsadlecki@wp.pl (P.S.); evi1710@op.pl (E.D.); walentowiczp@gmail.com (P.W.); grabiecm@cm.umk.pl (M.G.); 2 Department of Laboratory Medicine, L. Rydygier Collegium in Bydgoszcz, Nicolaus Copernicus University, M. Curie Skłodowskiej 9, Bydgoszcz 85-094, Poland; grazynaodes@interia.pl (G.O.-S.); asiapollak@wp.pl (J.S.)

**Keywords:** 25(OH)D, gestational hypertension, preeclampsia, gestational diabetes

## Abstract

Background: An association between the level of vitamin D and the risk of pregnancy-related complications remains unclear. The aim of this study was to examine concentrations of 25(OH) vitamin D in Polish women with normal pregnancies and pregnancies complicated by gestational hypertension, preeclampsia or gestational diabetes mellitus (GDM). Moreover, we analyzed an association between maternal serum 25(OH)D and the risk of gestational hypertension, preeclampsia and GDM. Material and Methods: The study included 207 pregnant women, among them 171 with pregnancy-related complications: gestational hypertension (*n* = 45), preeclampsia (*n* = 23) or GDM (*n* = 103). The control group consisted of 36 women with normal pregnancies. Concentrations of serum 25(OH)D were measured at admission to the hospital prior to delivery Results: Patients with hypertension did not differ significantly from the controls in terms of their serum 25(OH)D concentrations (18.20 vs. 22.10 ng/mL, *p* = 0.15). Highly significant differences were found in 25(OH)D concentrations of women with preeclampsia and the controls (14.75 vs. 22.10 ng/mL, *p* = 0.0021). GDM was not associated with significant differences in 25(OH)D concentration. A low level of 25(OH)D turned out to be associated with an increased risk of preeclampsia during pregnancy on both univariate and multivariate regression analysis, and was a significant predictor of this condition on ROC (receiver operating characteristic) analysis (AUC = 0.70, *p* < 0.01). Conclusions: 25(OH)D deficiency is common among pregnant Polish women. Low concentrations of 25(OH)D may play a role in the etiopathogenesis of preeclampsia. Routine assessment of the 25(OH)D level during pregnancy may be crucial for the identification of women at increased risk of preeclampsia.

## 1. Introduction

Vitamin 25(OH)D insufficiency/deficiency is currently recognized as a pandemic. Vitamin D insufficiency and deficiency occur in 9.3%–41.4% and 22.7%–90.3% of pregnant women, respectively [1]. Vitamin 25(OH)D deficiency results primarily from unawareness that moderate sun exposure constitutes the principal source of vitamin D3 in most humans [2]. The active form of vitamin D, i.e., 1,25(OH)_2_D, is now recognized as an important hormone playing a crucial role in skeletal homeostasis (classical effects) and exerting non-classical effects in various pathological and physiological conditions, such as cancer, arterial hypertension, cardiovascular diseases, brain development and immunomodulation [3,4,5,6].

Vitamin D3 is transported to the liver from the skin and intestines by vitamin D-binding protein (DBP) [7]. Here, it is hydroxylated to 25(OH)D, a major form of vitamin D found in the blood. Blood concentration of 25(OH)D reflects actual content of vitamin D in the human body [7]. The most active form of vitamin D, 1,25-dihydroxy-vitamin D, is synthesized in kidneys, as well as in other tissues and cells, e.g., parathyroid glands, prostate, macrophages, immune and reproductive cells [8]. The fact that 25(OH)D passes through the placenta to the fetus in early pregnancy, points to a pivotal role of appropriate dietary intake of vitamin D in pregnant women [9]. According to the Endocrine Society, insufficiency and deficiency of vitamin D are defined as concentrations of 25(OH)D equal to 20–30 ng/mL and <20 ng/mL, respectively. In turn, serum concentrations of 25(OH)D above 30 ng/mL are considered normal [9]. Deficiency of vitamin D is commonly diagnosed among pregnant women worldwide, and was shown to be associated with an increased risk of pregnancy-related complications [1].

Hypertensive disorders and gestational diabetes mellitus (GDM) are principal causes of morbidity and mortality in pregnant women, their fetuses and newborns [10,11]. Every tenth pregnant woman is diagnosed with gestational hypertension (GH), one of the most common complications and a leading cause of maternal mortality worldwide [12,13]. GH is the second most common cause of perinatal mortality due to hemorrhage in pregnant Polish women [14]. Depending on the study population and diagnostic criteria, prevalence of GH is estimated at 2%–7%. The risk of this complication was shown to increase with age, nulliparity, history of preeclampsia (PE) in previous pregnancies, diabetes mellitus and obesity [10,15]. PE is defined as hypertension (≥140/90 mmHg) and proteinuria, starting after 20 weeks of gestation [16]. Preeclampsia occurs in 5%–6% of pregnancies and constitutes one of the most common causes of perinatal complications, maternal, fetal and neonatal mortality [17,18,19].

GDM is another common complication of pregnancy. It is defined as glucose intolerance of any degree, which started or has been newly diagnosed in pregnancy. Depending on the study population and diagnostic criteria, GDM may affect up to 18% of pregnant women [20]. Principal risk factors of GDM include older age at conception and obesity. According to literature, a growing proportion of women decide to get pregnant at an older age, and the average age of getting pregnant is still increasing [21,22]. Metabolic disorders associated with GDM may negatively affect fetal development, which typically results in macrosomia, complicated labor, and perhaps also some postnatal complications related to fetal programming [23].

Although the biological role of vitamin D has been studied extensively, the consequences of its deficiency for maternal, fetal and neonatal health are still unclear [4,22,24]. The aim of this study was to determine serum concentrations of 25(OH)D in Polish women with uncomplicated pregnancies and pregnancies complicated by hypertension, preeclampsia and diabetes mellitus. Moreover, we analyzed an association between the concentration of 25(OH)D in maternal serum and the risk of hypertension, preeclampsia and diabetes mellitus.

## 2. Results

Concentrations of 25(OH)D within the range (≥30 ng/mL) was observed only in 10.8% patients. Insufficient concentrations of 25(OH)D (20–30 ng/mL) were found in 43.7% of pregnant women from the study group. However, deficiency of vitamin D (concentration 25(OH)D <20 ng/mL) was recognized in as many as 45.6% patients participating in the study. The results are presented in Table 1.

25(OH)D concentration did not differ significantly (*p* = 0.08) between groups considering seasons of taking blood (spring/summer vs. autumn/winter, 21.1 vs. 19.5 ng/mL, *p* = NS). Serum levels of 25(OH) (ng/mL) did not correlate significantly with pregnancy duration, neonatal birth weight and pH of the umbilical cord blood (data not shown).

25(OH)D concentration did not differ significantly (*p* = 0.0623) between the group of patients with hypertension and the control group (18.20 vs. 22.10 ng/mL *p* = 0.1481). However, there were high significant differences in 25(OH)D concentration between the group of women with preeclampsia and the control group (14.75 vs. 22.10 ng/mL, *p* = 0.0021). There were no differences in 25(OH)D concentration between the group with GDM G1 (20.80 ng/mL), the group with GDM G2 (22.60 ng/mL) and the control group (22.10 ng/mL) (*p* = 0.9207). The results are presented in Table 2.

ROC analyses were performed to assess the relationship between 25(OH)D and the incidence of gestational hypertension (GH), preeclampsia (PE) and gestational diabetes mellitus (GDM) (Table 3).

**Figure 1:**
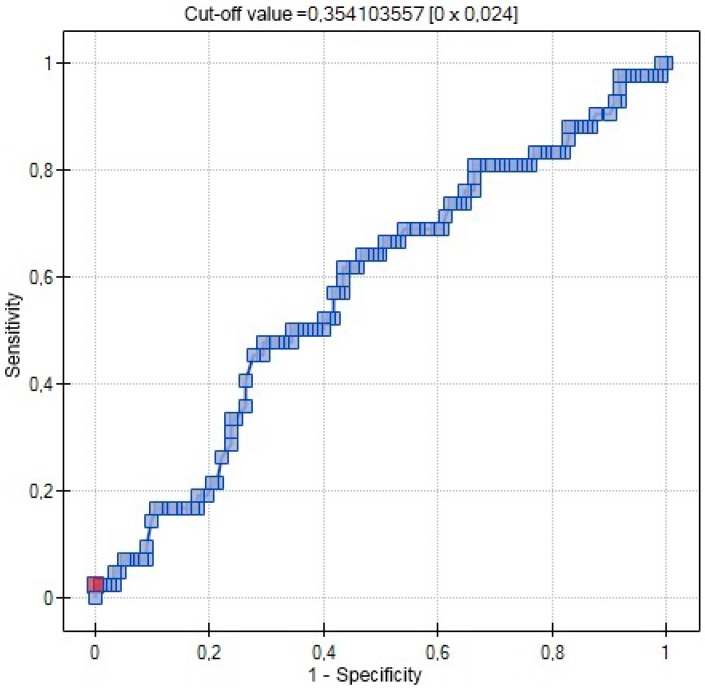
ROC curve relationship between 25(OH)D concentrations (ng/mL) and occurrence of gestational hypertension (red point—cut-off value).

In ROC analysis 25(OH)D was not a significant variable of the incidence of gestational hypertension (Figure 1).

**Figure 2:**
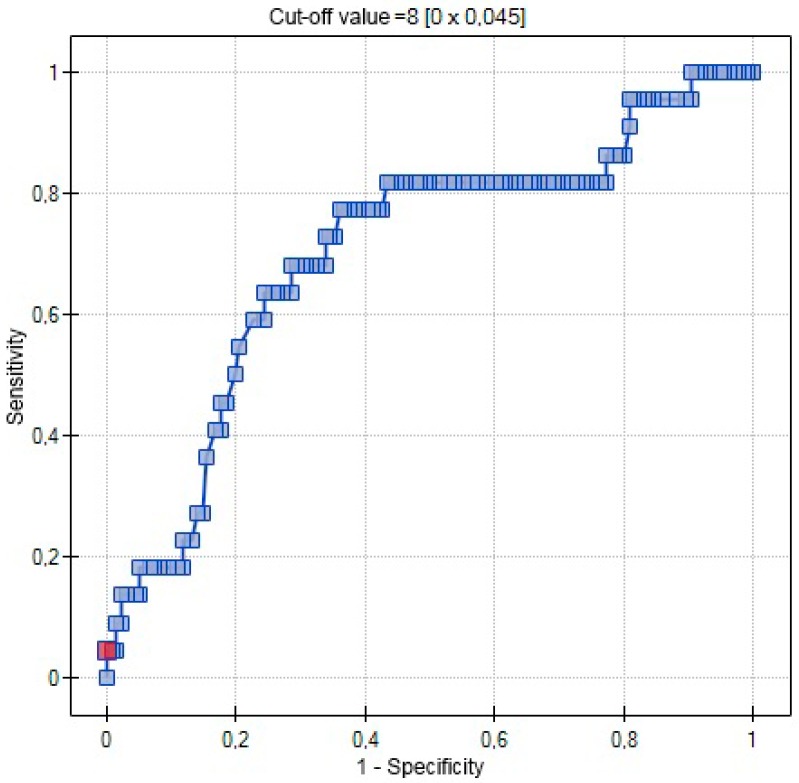
ROC curve-relationship between 25(OH)D concentrations (ng/mL) and occurrence of preeclampsia (AUC 0.7029), (red point—cut-off value).

In the ROC analysis serum 25(OH)D was a significant destimulator of the incidence of preeclampsia (*p* = 0.0023), with the area under the ROC curve (AUC) equal to 70.3%. (Figure 2).

**Figure 3:**
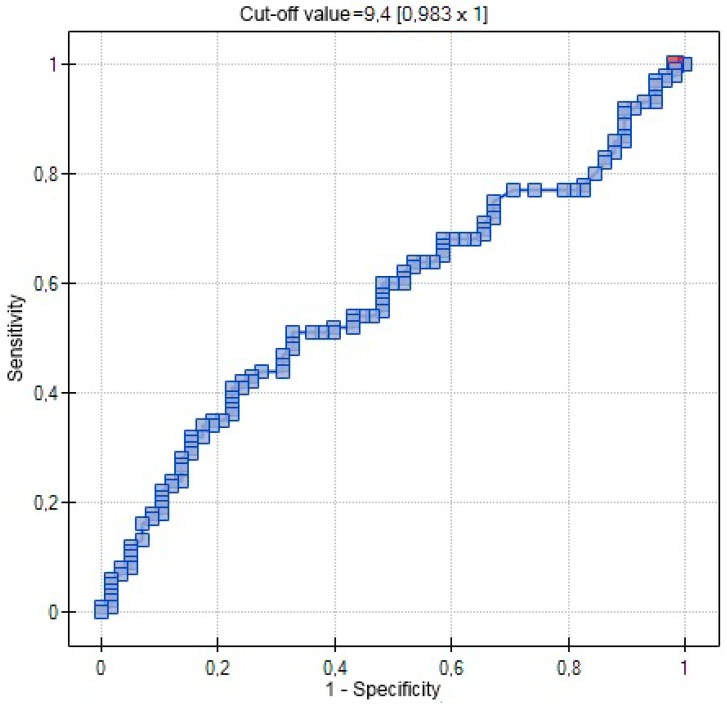
ROC curve-relationship between 25(OH)D concentrations (ng/mL) and occurrence of diabetes (AUC 0.5721) (red point—cut-off value).

In ROC analysis 25(OH)D was not a significant variable influencing the incidence of diabetes (Figure 3).

Univariate and multivariate logistic regression analysis was conducted to verify if the effects of serum 25(OH)D levels on the incidence of preeclampsia are independent of other established risk factors (age >35, primiparity) of these conditions (Table 4).

Serum 25(OH)D (ng/mL) and primiparity were identified as an independent predictor of preeclampsia on both univariate and multivariate logistic regression analyses. Low serum 25(OH)D levels and primiparity increased the risk of preeclampsia.

## 3. Discussion

Only recently have we started to understand the developmental origins of diseases and the influence of perinatal environment on lifelong health. Despite widespread deficiency of vitamin D among pregnant women worldwide, its role in pregnancy has largely been ignored [25,26,27]. Only 10.8% of our patients presented sufficient serum concentrations of 25(OH)D (≥30 ng/mL), and the diagnostic criteria for vitamin D insufficiency and deficiency (i.e., 25(OH)D 20–30 ng/mL or <20 ng/mL) were satisfied by 43.7% and 45.6% of the study subjects, respectively. One potential explanation for such a high prevalence of vitamin D insufficiency/deficiency among pregnant Polish women is a relatively low number of sunny days in our country. Furthermore, most pregnant women are known to avoid sun exposure. Finally, low serum concentrations of 25(OH)D observed in our series might also result from an inadequate supplementation of this vitamin. Our findings are consistent with the results of another study conducted in a central European population, which also documented a high prevalence of vitamin D deficiency [23].

Taking into account inconclusive results of cross-sectional and cohort studies analyzing the association between vitamin D status and occurrence of pregnancy pathologies [28,29], the aim of our study was to asses 25(OH)D status in women with gestational hypertension (GH). Our study showed that pregnant women with GH and healthy controls did not differ significantly in terms of their 25(OH)D concentrations (18.20 ng/mL vs. 22.10 ng/mL, *p* = 0.1481), and serum 25(OH)D has not been identified as a significant predictor of GH on either logistic regression analysis or ROC analysis. These observations are consistent with the results published by Bärebring et al. [13] who demonstrated that vitamin D status correlates significantly with blood pressure during the first trimester and gestational systolic blood pressure trajectory, but not with the prevalence of GH. The relationship between vitamin D status and GH has also been a subject of other studies. Van Weert et al. analyzed associations between vitamin D status, blood pressure at the onset of pregnancy and occurrence of a mid-pregnancy decrease in the latter parameters. Moreover, the authors searched for a potential mechanism explaining the association between vitamin D status and pregnancy-related hypertensive disorders. Similar to our study, they found no association between vitamin D status, prevalence of GH, blood pressure at early gestation and mid-pregnancy decrease in this parameter [30]. Similar findings were reported by Powe et al., who did not observe a relationship between the level of 25(OH)D, systolic and diastolic blood pressure. The same study did not document significant differences in 25(OH)D concentrations in women who developed hypertension later in pregnancy and those who did not [31]. In contrast, Burris et al. showed that the risk of GH increased by 33% per each additional 25 nmol/L of serum 25(OH)D. Although this observation was interpreted as a random finding, women with higher 25(OH)D levels were still at increased risk of gestational hypertension [29]. Perhaps, women who already had been diagnosed with GH were more compliant with the recommended multivitamin intake, which was eventually reflected by their higher serum concentrations of 25(OH)D [29].

In our opinion, some discrepancies between the results of previous studies may result from lack of adjustment for vitamin D supplementation and season of the blood sampling. Consequently, the effect of serum 25(OH)D on the occurrence and clinical presentation of gestational hypertension still needs to be elucidated, in line with Hill’s criteria [32]. Hence, we postulate to conduct a well-designed randomized study to assess the role of vitamin D in the etiopathogenesis of GH.

In this study, we found evident, statistically significant differences in serum concentrations of 25(OH)D in women with PE and healthy controls (14.75 ng/mL vs. 22.10 ng/mL, *p* = 0.0021). Moreover, a significant association between serum 25(OH)D and PE (*p* = 0.0023) was documented on ROC analysis, with the area under the ROC curve (AUC) equal to 70.3%. Consequently, we conducted a logistic regression analysis to verify if the effects of serum 25(OH)D on the incidence of preeclampsia are independent of other established risk factors of this condition (age > 35 years, primiparity). Both low serum concentration of 25(OH)D (ng/mL) and primiparity were identified as independent predictors of PE (*p* = 0.0019) on multivariate logistic regression analysis. Our results presented here, obtained in a cohort of pregnant Polish women, are consistent with previously reported findings [32]. According to Barebring et al., at least a 30 nmol/L increase in serum 25(OH)D concentration is associated with lower odds of PE, regardless of vitamin D status in early pregnancy [13]. Similar to our study, these authors found an inverse association between the occurrence of PE and serum concentration of 25(OH)D during the third trimester. In our study, we determined vitamin D status at a single time point, shortly before delivery. It would be interesting to measure vitamin D levels throughout the whole pregnancy. Previous studies demonstrated that the level of 25(OH)D in the first trimester was not related to the risk of PE [13]. Consequently, vitamin D level in early pregnancy is less likely to play a major role in placental development, and it is later increased in this parameter which may prevent the development of PE [13]. The mechanism through which a low concentration of vitamin D contributes to preeclampsia is still unclear. 1,25(OH)2D was postulated to modulate immunological tolerance during pregnancy, which may explain its role in PE pathogenesis [33,34].

Another potential explanation for the association between serum 25(OH)D and occurrence of PE may stem from the fact that the latter condition is associated with impaired placentation and maternal endothelial dysfunction [16]. Preeclampsia in patients with vitamin D insufficiency may be linked to a low concentration of the vascular endothelial growth factor (VEGF) and high levels of inflammatory cytokines; expressions of these factors are under a strong influence of vitamin D [35,36,37,38].

Both a recent meta-analysis and several observational studies showed a significant association between vitamin D deficiency and increased risk for preeclampsia [39,40,41]. Similar to our study, Pena et al. demonstrated that preeclamptic women more often present a 25(OH)D deficiency shortly prior to delivery [42]. Also, Mohaghegh et al. reported significantly lower mean levels of 25(OH)D in preeclamptic women [43]. Some evidence suggests that preeclampsia may be associated with a decrease in placental and fetal concentrations of vitamin D, and placental dysfunction plays an important role in the pathogenesis of PE [44]. Appropriate development and function of the placenta are prerequisites of normal pregnancy outcome. During pregnancy, 1,25(OH)2D3 may be synthesized not only by kidneys but also by trophoblast. The human placenta and decidua are capable of producing and secreting 1,25(OH)2D3, and some studies analyzed mRNA expression of 1α-hydroxylase and receptor VDR [45]. Although preeclampsia has been linked to maternal vitamin D insufficiency/deficiency, still little is known on potential differences in placental metabolism of this vitamin in uncomplicated and preeclamptic pregnancies [32].

Owing the potential contribution of an altered vitamin D metabolism to diabetes mellitus, we compared serum concentrations of 25(OH)D in patients with type 1 and 2 GDM (GDM G1 and GDM G2) and healthy controls. The analyzed groups did not differ significantly in terms of their 25(OH)D concentrations (20.80 ng/mL vs. 22.60 ng/mL vs. 22.10 ng/mL, *p* = 0.9207), and 25(OH)D was not identified as a significant predictor of GDM on ROC analysis.

Our findings are consistent with the results of the study conducted by Farrant et al. [46] who also did not find a link between the concentration of 25(OH)D and prevalence of diabetes mellitus among 559 pregnant women from India [46]. Also, Loy et al. did not observe a relationship between 25(OH)D inefficiency/deficiency and GDM [47]. In the B.A.B.Y. study, an increase in serum concentration of 25(OH)D was associated with a greater risk for gestational diabetes mellitus, but only among Hispanic women [48]. Similar to our study, Pleskacova et al. did not observe significant differences in vitamin D levels among pregnant women and healthy controls; nevertheless, the prevalence of a vitamin D deficiency in both groups was quite high [23]. However, published evidence regarding the relationship between vitamin D level and occurrence of GDM is inconclusive. Furthermore, a direct link between glucose metabolism and vitamin D pathways has been identified. 1,25(OH)2D may induce insulin secretion and was shown to contribute to a decrease in insulin resistance; therefore, low levels of this vitamin may play a role in the development of GDM [32]. A meta-analysis conducted by Zhang et al. demonstrated that deficiency of vitamin D may predispose to GDM. Consequently, these authors recommended to conduct a well-designed randomized controlled trial to examine the exact role of vitamin D supplementation in the prevention of GDM [49]. To summarize, the link between vitamin D and glucose metabolism requires further studies on larger populations of pregnant women.

## 4. Materials and Methods

Initially, a total of 244 patients were enrolled, but then women with multiple pregnancies (*N* = 5), intrauterine growth restriction (*N* = 7), premature rupture of membranes (*N* = 5) and hypothyroidism were excluded (*N* = 9), as well as the participants with heart problems and other co morbidities (*N* = 11). The study group was recruited from patients hospitalized at the Department of Obstetrics and Gynecology in University Hospital in Bydgoszcz. A total of 207 women, aged 19–40 years old, in singleton pregnancy, were included in the study (all Caucasian of Polish nationality, from Kuyavian-Pomeranian Region). The research was conducted in 2013–2015 at the Department of Obstetrics and Gynecology, Ludwik Rydygier Collegium Medicum in Bydgoszcz, Nicolaus Copernicus University of Torun.

A total of 45 women developed arterial hypertension during pregnancy, and 23 were diagnosed with preeclampsia. GDM was detected in 103 patients. In 84 patients GDM G2 occurred and in 19 patients GDM G1. Patients were stratified according to GDM type, G1 or G2, in line with the White classification [50]. Type A1 (corresponding to GDM G1) was defined as abnormal oral glucose tolerance test (OGTT), but normal blood glucose levels during fasting and one/two hours after a meal; diet modification was sufficient to control glucose levels. Type A2 (corresponding to GDM G2) was defined as abnormal OGTT compounded by abnormal glucose levels during fasting and/or after a meal; additional therapy with insulin or other medications was required. Patients who presented both arterial hypertension and GDM were excluded from the study. Gestational hypertension was defined as a systolic blood pressure >140 mmHg or diastolic blood pressure >90 mmHg on two or more measurements at least six hours apart, occurring after 20 weeks of gestation, without concomitant proteinuria. Preeclampsia was defined as the onset of hypertension (systolic blood pressure ≥140 mmHg or diastolic blood pressure ≥90 mmHg) in a previously normotensive woman, and proteinuria (at least 0.3 g of protein in a 24 h urine sample) without a concomitant urinary tract infection. Gestational diabetes was detected on the basis of a 75 g glucose tolerance test conducted between 24 and 28 weeks of gestation. All patients were in appropriate-for-gestational age (AGA) pregnancies. Fetuses were defined as AGA during pregnancy by an ultrasound scan. Patients, after in vitro fertilization, were excluded from the study, as were women with any other concomitant diseases. In all patients, serum 25(OH)D was determined during the hospital stay, up to seven days before delivery. Women presenting abnormal blood pressure were additionally examined for the severity of hypertension and presence of preeclampsia. Patient age, parity, body mass index (BMI), gestational age at delivery, route of delivery, birth weight and pH of the umbilical cord blood were analyzed. The control group included 36 women with uncomplicated pregnancies, normal arterial blood pressure and glucose concentrations. Baseline characteristics of study participants are presented in Table 5.

### 4.1. Methods

Blood samples of 10-mL were obtained from the cubital vein and immediately separated by centrifugation. The serum was stored at −80 °C until analysis. Subsequently, serum 25(OH)D concentration was determined by ELISA (25-Hydroxy Vitamin D EIA), Immunodiagnostic Systems Ltd. in Department of Laboratory Diagnostics, Ludwik Rydygier Collegium Medicum, Nicolaus Copernicus University in Torun; sensitivity 2.0 ng/mL (5 nmol/L), assay measured range was 2.7–152 ng/mL (6.8–380 nmol/L). A certified reference material (NIST Standard Reference Material (SRM) 972) was used for vitamin D measurement. The method remained under RIQAS control, and the results of the assay are within the reference range for this control. The inter- and intra assay variabilities were 5.3% and 4.6%. Concentration of 25(OH)D <10 ng/mL (<25 nmol/L) was defined as deficiency, 10–29 ng/mL (25–74 nmol/L) has been accepted as insufficiency, and sufficient when concentrations were between 30–100 ng/mL (75–250 nmol/L).

### 4.2. Statistical Analyses

All statistical analyses were performed using the PQStat version 1.6 (StatSoft, Tulsa, OK, USA). The Mann-Whitney *U*-test and the Kruskal-Wallis test withpost-hoc Dunn’s testwere applied to compareconcentrations of 25(OH)D (ng/mL) between the groups. Relationships between serum concentrations of 25(OH)D (ng/mL),patient age, pH of the umbilical cord blood and birth weight were assessed on the basis of Spearman’s rank correlation coefficient values. Prediction of hypertension, preeclampsia and diabetes (GDM G1 and GDM G2) were analyzed with univariate and multivariate models of logistic regression. The influence of the concentration of the 25(OH)D on the appearance of hypertension and preeclampsia were also analyzed with ROC curves. *p*-Value <0.05 was considered statistically significant and *p*-value <0.01 was considered statistically highly significant.

The study design was approved by The Ethical Committee, of The LudwikRydygier Collegium Medicum in Bydgoszcz (decision No. KB 502/2013, 2 December 2013). All participants have provided the informed, written consent.

## 5. Conclusions

In conclusion, both the results of previous studies and our findings presented here, documenting the link between maternal deficiency of vitamin D and the increased risk of preeclampsia, point to the important role of an appropriate level and adequate metabolism of this vitamin in pregnancy. However, we are well aware of the potential limitations of this study. The relatively small size of the sample did not enable us to formulate any ultimate conclusions. Consequently, larger studies are needed to confirm the findings presented here and to explore their underlying etiopathogenic mechanisms. It would be necessary to get detailed information on vitamin D and calcium intake, as well as on patients’ habits (outdoor activities and sunbathing). Available evidence is still insufficient to confirm a causative link between vitamin D and pregnancy disorders in line with Hill’s criteria. Nevertheless, our study demonstrated a high prevalence of 25(OH)D deficiency/insufficiency among pregnant Polish women.

## Figures and Tables

**Table 1:** Concentration of 25(OH)D (ng/mL) in the study group.

25(OH)D Concentration (ng/mL)	Study Group (*N* = 171)	Control Group (*N* = 36)
Sufficient concentration: 25(OH)D ≥ 30	18 (10.8%)	11 (30.5%)
Insufficientconcentration: 25(OH)D: 20–30	75 (43.7%)	15 (41.7%)
Deficiency: 25(OH)D < 20	78 (45.6%)	10 (27.8%)

**Table 2:** Serum concentrations of 25(OH)D (ng/mL) in various subsets of patients (Q1—lower quartile; Q3—upper quartile; SD—standard deviation; NS—non-significant; GH—gestational hypertension; PE—preeclampsia; GDM—gestational diabetes mellitus).

25(OH)D ng/mL	*N*	Mean	SD	Minimum	Q1	Median	Q3	Maximum	Mann-Whitney *U*-Text
GH	45	19.01	7.36	8.00	13.70	18.20	24.30	37.50	NS
Controls	36	21.65	5.13	14.60	16.95	22.10	24.90	32.30	
PE	23	21.65	5.13	14.60	16.95	22.10	24.90	32.30	*Z* = 3.0759 *p* = 0.0021
Controls	36	21.65	5.13	14.60	16.95	22.10	24.90	32.30	
GDM	103	21.99	7.43	9.40	15.50	22.10	27.30	47.10	NS
Controls	36	21.65	5.13	14.60	16.95	22.10	24.90	32.30	

**Table 3:** Relationships between serum concentrations of 25(OH)D and the incidence of pregnancy-induced hypertension (PIH); gestational hypertension (GH); preeclampsia (PE); gestational diabetes mellitus (GDM); results of ROC (receiver operating characteristic) analysis.

Parameter	GH	PE	GDM
AUC	0.5767	0.7029	0.5721
SE (AUC)	0.0509	0.0631	0.0462
−95% CI	0.4769	0.5792	0.4817
+95% CI	0.6765	0.8265	0.6626
*Z*-statistic	1.4806	3.0486	1.5096
*p*	0.1387	0.0023	0.1311
Cut-off value	0.3541	8	9.4

**Table 4:** Results of univariate and multivariate logistic regression analysis examining the effects of serum 25(OH)D levels (ng/mL), age and primiparity on the incidence of preeclampsia.

Parameter	*b*-Coefficient	*p*-Value	Odd Ratio	+95% CI	+95% CI
Intercept	0.4965	0.5113	1.6429	0.3735	7.2268
25(OH)D (ng/mL)	−0.1208	0.0033	0.8862	0.8177	0.9605
Intercept	0.0467	0.9703	1.0478	0.0899	12.2172
Age > 35 years old	−0.0644	0.1298	0.9377	0.8627	1.0191
Intercept	−2.6509	<0.0001	0.0706	0.0308	0.1615
Primiparity	1.3410	0.0077	3.8228	1.4260	10.2478
Intercept	0.3649	0.8328	1.4403	0.0487	42.5836
25(OH)D (ng/mL)	−0.1170	0.0053	0.8896	0.8195	0.9658
Age > 35 years old	−0.0267	0,5818	0.9737	0.8856	1.0706
Primiparity	1.3980	0.0166	4.0469	1.2893	12.7030

**Table 5:** Baseline characteristics of the study participants. NS: not significant; BMI: body mass index; P: *p*-value.

Parameter	Study Group *N* = 171	Control Group *N* = 36	P
Age (years)	29.6 ± 5.2	29.4 ± 4.9	NS
BMI (kg/m^2^)	27.8 ± 2.2	26.9 ± 2.4	NS
Parity	1.9 ± 1.1	1.8 ± 1.0	NS
pH of umbilical artery	7.35 ± 0.09	7.35 ± 0.07	NS
BMI (kg/m^2^)	24.8 ± 2.0	25.2 ± 2.5	NS
Pregnancy (weeks)	38 ± 2.96	40 ± 1.08	NS
Caesarian sections %	43.3	37.6	NS
Weight of newborn (g)	3340 ± 680	3590 ± 430	NS
